# Native and IgE-primed rat peritoneal mast cells exert pro-inflammatory activity and migrate in response to yeast zymosan upon Dectin-1 engagement

**DOI:** 10.1007/s12026-021-09183-7

**Published:** 2021-03-11

**Authors:** Paulina Żelechowska, Ewa Brzezińska-Błaszczyk, Sylwia Różalska, Justyna Agier, Elżbieta Kozłowska

**Affiliations:** 1 Department of Experimental Immunology, Faculty of Health Sciences, Medical University of Lodz, Pomorska 251, 92-213 Lodz, Poland; 2 Department of Industrial Microbiology and Biotechnology, Faculty of Biology and Environmental Protection, University of Lodz, Banacha 12/16, 90-237 Lodz, Poland

**Keywords:** Fungal infection, Host defense, Immune response, Immune mediators, Mast cell

## Abstract

Mast cells (MCs) play an essential role in host defense, primarily because of their location, their ability to pathogen destruction via several mechanisms, and the pattern recognition receptors they express. Even though most data is available regarding MC activation by various bacteria- or virus-derived molecules, those cells’ activity in response to constituents associated with fungi is not recognized enough. Our research aimed to address whether *Saccharomyces cerevisiae*-derived zymosan, i.e., β-(1,3)-glucan containing mannan particles, impacts MC activity aspects. Overall, the obtained results indicate that zymosan has the potential to elicit a pro-inflammatory response of rat peritoneal MCs. For the first time ever, we provided evidence that zymosan induces fully mature MC migration, even in the absence of extracellular matrix (ECM) proteins. Moreover, the zymosan-induced migratory response of MCs is almost entirely a result of directional migration, i.e., chemotaxis. We found that zymosan stimulates MCs to degranulate and generate lipid mediators (cysLTs), cytokines (IFN-α, IFN-β, IFN-γ, GM-CSF, TNF), and chemokine (CCL2). Zymosan also upregulated mRNA transcripts for several cytokines/chemokines with pro-inflammatory/immunoregulatory activity. Moreover, we documented that zymosan activates MCs to produce reactive oxygen species (ROS). Lastly, we established that the zymosan-induced MC response is mediated through activation of the Dectin-1 receptor. In general, our results strongly support the notion that MCs contribute to innate antifungal immunity and bring us closer to elucidate their role in host-pathogenic fungi interactions. Besides, provided findings on IgE-sensitized MCs appear to indicate that exposure to fungal zymosan could affect the severity of IgE-dependent disorders, including allergic ones.

## Introduction

Mast cells (MCs) are long-lived cells that have a widespread distribution in the entire body’s connective tissue. The well-recognized feature of these cells is the capability of secreting de novo-generated mediators and rapidly releasing preformed products stored in cytoplasmic granules [1, 2]. That is why MCs exert a broad spectrum of effector functions through direct or indirect actions on cells or tissues located in their close proximity. MCs participate in various health- and/or disease-associated processes, ranging from homeostasis maintenance to FcεRI-mediated allergic reactions and carcinogenesis [3–5]. Undoubtedly, MCs orchestrate a cascade of various events associated with acute and chronic inflammation [6]. They also play a critical regulatory role in eliciting both innate and adaptive immune responses [7].

MCs’ strategic position at the host-environment interfaces causes that they are among the first cells to interact with various invading microbes and trigger a response against them [8–10]. MCs may combat and initiate the clearance of pathogens by releasing inflammatory mediators and antimicrobial peptides, reactive oxygen species (ROS) production, phagocytosis, extracellular trap (ECM) formation, and pathogen antigen presentation through class I and II MHC molecules [11, 12]. Undoubtedly, MCs may act as effectors of host defense through their ability to detect distinct pathogen-associated molecular patterns (PAMPs) by specific molecules termed pathogen recognition receptors (PRRs). Among them, the best studied are Toll-like receptors (TLRs), which recognize mainly bacterial membrane components, but also they can sense bacterial DNA and, same as RIG-I-like receptors (RLRs), viral dsRNA, and ssRNA [13, 14]. MCs also express NOD-like receptors (NLRs), which recognize intracellularly encountered microbial motifs [13]. Among PRRs expressed on MCs, there are molecules responsible for sensing carbohydrate particles present in the fungal cell wall. They include Dectin-1, mannose receptor (MR), and Mincle from the C-type lectin receptor (CLR) family and some representatives from TLR class [13]. Even though most data is available regarding MC activation by various bacteria- or virus-derived antigens, those cells’ activity in response to constituents associated with fungal cell wall is not recognized enough.

In general, the fungal cell wall is a rigid structure composed of various types of linear and branched polysaccharides, glycoproteins, proteins, or lipids that are organized in at least two layers. The outer layer of the fungal cell wall mainly consists of mannose homopolymers, that is, mannans and mannoproteins. The cell wall’s inner layer includes chitin and glucans, and 50–60% of the dry weight of this structure is made up of glucan with β-1,3-glycosidic linkages [15]. Hence, β-(1,3)-glucan is principally involved in the interplay between pathogenic fungi and the host immune system. β-(1,3)-glucan influences immune cell activity affecting their phagocytic and cytotoxic activity, cytokine/chemokine, as well as ROS and nitrogen intermediate (NO) production [16–18]. Our study’s objective was to address whether *Saccharomyces cerevisiae*-derived zymosan, i.e., β-(1,3)-glucan containing mannan particles, impact some aspects of peritoneal MC activity. As yet, the effect of zymosan on MC degranulation and synthesis of several mediators was explored only on cord blood-derived MC (CBMC) [19, 20] and murine bone marrow-derived MC (BMMC) model [21, 22]. The current study evaluates zymosan’s influence on MC migration, degranulation, and generation and/or release of de novo-synthesized mediators/cytokines/chemokines. The contribution of signaling molecules to zymosan-mediated MC responses was also estimated.

## Materials and methods

### Reagents

Dulbecco’s Modified Eagle Medium (DMEM) was purchased from Biowest (Riverside, MO, USA). Hank’s Balanced Salt Solution (HBSS), NaHCO_3_, fetal calf serum (FCS), gentamicin, and glutamine were obtained from GIBCO (Gaithersburg, MD, USA). NaCl, KCl, MgCl_2_, CaCl_2_, *N*-2-hydroxyethylpiperazine-*N’*-2-ethanesulfonic acid (HEPES), NaOH, glucose, HCl, *o*-phthalaldehyde (OPT), compound 48/80, calcium ionophore A23187, Percoll®, hematoxylin, toluidine blue, trypan blue, bovine serum albumin (BSA), zymosan from *Saccharomyces cerevisiae*, curdlan from *Alcaligenes faecalis*, laminin from human placenta, phosphate-buffered saline (PBS), ethanol 99.8%, PCR grade water, and laminarin from *Laminaria digitate* were obtained from Sigma-Aldrich (St. Louis, MO, USA). Resiquimod (R848) and Syk kinase inhibitor R406 were purchased from InvivoGen (San Diego, CA, USA). Recombinant rat TNF was obtained from R&D Systems (Minneapolis, MN, USA). RNeasy® Mini Kit was obtained from Qiagen (Valencia, CA, USA). High-Capacity cDNA Reverse Transcription Kit was purchased from Applied Biosystems (Foster City, CA, USA). SsoAdvanced™ Universal SYBR® Green Supermix was obtained from Bio-Rad Laboratories (Hercules, CA, USA). Primers were purchased from IBB PAN (Warsaw, Poland). Mouse anti-rat IgE monoclonal IgG1 antibodies (anti-IgE) were purchased from AbD Serotec (Oxford, UK). Cysteinyl leukotriene (cysLT) ELISA kit was obtained from Cayman Chemical (Ann Arbor, MI, USA). Rat interferon (IFN)-α and IFN-β specific immunoassay kits were purchased from Wuhan Fine Biotech Co. (Wuhan, China). Rat IFN-γ, tumor necrosis factor (TNF), granulocyte-macrophage colony-stimulating factor (GM-CSF), CCL2, and CCL3 specific ELISA kits were obtained from Biorbyt Ltd. (Cambridge, UK). CellROX™ Green Reagent and rat IgE purified from ascites induced by the myeloma cell line IR 162 (Cat. 02-9788) were purchased from Invitrogen (Carlsbad, CA, USA). Human plasma fibronectin purified protein was purchased from Merck Millipore (Billerica, MA, USA). The 48-well Boyden microchamber and the 8-μm-pore-size polycarbonate filters were purchased from Neuro Probe (Gaithersburg, MD, USA).

### MC collection

Wistar adult female albino rats weighing 200–250 g were used. To collect MCs, lavage of peritoneal cavities with 50 ml of 1% HBSS supplemented with 0.015% NaHCO_3_ was conducted. Subsequently, the cell suspension was gently removed from the peritoneal cavity, centrifuged (150×*g*, 5 min, 20°C), and washed two times in complete (c)DMEM containing DMEM (supplemented with 10% of FCS, 2 mM of glutamine, and 10 μg/ml of gentamicin). To prepare a pure fraction of MCs, peritoneal cells’ suspension was resuspended in 72.5% isotonic Percoll and centrifuged (190×*g*, 15 min, 20°C). The upper cell layer was removed, and a double-wash procedure of pelleted MCs in cDMEM was carried out by centrifugation (150×*g*, 5 min, 20°C). Next, MCs were counted and resuspended in an appropriate volume of medium for rat MCs, containing 137 mM NaCl, 2.7 mM KCl, 1 mM CaCl_2_, 1 mM MgCl_2_, 10 mM HEPES buffer, 5.6 mM glucose, and 1 mg/ml BSA (for histamine release assay, ELISA technique, and ROS generation measurement) or cDMEM (for quantitative RT-PCR and migration assay) to get the MC count of 1.5 × 10^6^ cells/ml. To acquire appropriate MC density and number of samples in a given type of experiment, the proper number of animals was used. Cell viability (which exceeded 98%) was determined using the trypan blue exclusion method, and MC purity (>98%) was evaluated by the presence of metachromatic granules using staining with toluidine blue. The MC collection procedure from rats was reviewed and approved by the Local Ethics Committee for Experiments on Animals in Lodz.

### IgE-mediated priming of MCs

For some set of experiments, MCs coated with IgE were prepared. To this end, MCs suspended in cDMEM were incubated with rat IgE at 1 μg/ml for 1 h at 37°C. To remove unbound IgE after incubation, MCs’ double-wash procedure was carried out by centrifugation (150×*g*, 5 min, 20°C). Next, IgE-coated MCs were resuspended in a suitable volume of medium for rat MCs (for histamine assessment, cysLT analysis, and ROS measurement) or cDMEM (for qRT-PCR and migration assay).

### Migration assay

The migration of native and IgE-sensitized MCs was performed in a 48-well Boyden microchamber using standard 8-μm-pore-sized polycarbonate filters. Where noted, laminin- or fibronectin-coated filters were used. These were prepared by soaking filters overnight in fibronectin or laminin (100 μg/ml) and air-drying for at least 1 h before use. A volume of 30 μl of zymosan at final concentrations of 0.01, 0.1, 1, 10, or 50 μg/ml, TNF at a final concentration of 0.05 pg/ml (positive control), or medium alone (control spontaneous MC migration) was placed in the lower wells of the microchamber. The bottom wells were covered with a micropore filter, and 50 μl of the MC suspension was loaded into the top wells. Then, the microchamber was placed in an incubator containing 5% CO_2_ humidified atmosphere for 3 h. After the incubation, the upper part of the Boyden microchamber (top wells) was removed, and the upper surface of the polycarbonate membrane was wiped carefully with the use of a rubber scraper. The polycarbonate filter was then fixed in 99.8% ethanol, stained with hematoxylin (for 10 min), rinsed in distilled water, and mounted on a microscope slide. MC migratory response was examined by counting the number of cells that had traversed the membrane and attached to its bottom surface. The number of MCs in 10 high-power fields (HPF) was counted in each assay, and the results are expressed as a percentage of control spontaneous migration (referred to as 100%).

### Checkerboard analysis

Checkerboard analysis was conducted to examine whether zymosan-induced MC migration was a chemotactic or chemokinetic response. To this end, different concentrations of zymosan were added to the top and bottom compartments of the Boyden microchamber. Migration assay was carried out as described above, using uncoated micropore filters. Chemotactic mobility was recognized whenever a positive gradient of the chemoattractant was observed. Chemokinesis was identified when the zymosan was present in both the bottom and top compartments at the same concentrations (equivalent concentrations) or when the zymosan was present in the upper compartments of the microchamber (reversed gradient).

### Determination of histamine release

Native and IgE-coated MCs suspended in a medium for rat MCs were incubated with zymosan at final concentrations of 0.01, 0.1, 1, 10, or 50 μg/ml, compound 48/80 at a final concentration of 5 μg/ml (positive control), or medium alone (spontaneous histamine secretion) in a water bath for 30 min at 37°C with continuous stirring. For time-course experiments, native MCs were challenged with zymosan at a final concentration of 50 μg/ml for 0, 1, 3, 5, 10, or 30 min. The reaction was stopped by adding 1.9 ml of cold medium to each sample. Afterward, the cell suspensions were centrifuged (253×*g*, 5 min, 4°C), and the supernatants were transferred directly into a new set of tubes. Two milliliter of distilled water was added to each sample with the cell pellet. The histamine content was analyzed in cell pellets (residual histamine) and supernatants (released histamine) using OPT by a spectrofluorometric method with an excitation wavelength of 360 nm and a fluorescence wavelength of 450 nm. Histamine secretion was expressed as a percentage of the total cellular content of this amine.

### CysLT synthesis measurement

Native and IgE-coated MCs suspended in the medium for rat MCs were incubated with zymosan at final concentrations of 0.01, 0.1, 1, 10, or 50 μg/ml, calcium ionophore A23187 at a final concentration of 5 μg/ml (positive control), or medium alone (spontaneous cysLT release) in a water bath for 1 h at 37°C with continuous stirring. Afterward, the cell suspensions were centrifuged (150×*g*, 5 min, 20°C), and the supernatants were transferred directly into a new set of tubes. CysLT levels in supernatants were measured by commercial ELISA kit according to the manufacturer’s instruction. The assay’s sensitivity was 20 pg/ml.

### qRT-PCR

The qRT-PCR assay was used to evaluate zymosan-induced cytokine/chemokine mRNA expression levels in MCs. To this end, native and IgE-coated MCs suspended in cDMEM were incubated with zymosan at a final concentration of 50 μg/ml for 2 h at 37°C in an incubator containing 5% CO_2_ humidified atmosphere. Afterward, the total RNA from MCs was isolated using the RNeasy® Mini Kit. According to the manufacturer’s protocol, the first-strand cDNA synthesis was carried out using a High-Capacity cDNA Reverse Transcription Kit. The PCR reaction mixture contained 5 μl of SsoAdvanced™ Universal SYBR® Green Supermix, 2 μl of primers (500 nM), 1 μl of cDNA, and 2 μl of PCR-grade water. Primer sequences are listed in Table 1. The reactions were performed with the use of the CFX Connect™ Real-Time PCR Detection System (Bio-Rad Laboratories, Hercules, CA, USA). PCR cycle conditions involved an initial denaturation step (95°C for 30 s) followed by 40 cycles of denaturation (95°C for 10 s) and then annealing/extension (60°C for 10 s). The fold changes in the expression were determined by the _ΔΔ_Ct method using Bio-Rad CFX Maestro™ Software. The reference gene rat *Actb* was used to normalize the expression of cytokine/chemokine mRNAs. Unstimulated specimens were used as calibration samples.

**Table 1:** List of primer sequences used for qRT-PCR analysis in this study

Gene name	Primer sequence (5′-3′)
*Actb*	Forward: TCTGTGTGGATTGGTGGCTCTA Reverse: CTGCTTGCTGATCCACATCTG
*Il1b*	Forward: CACCTCTCAAGCAGAGCACAG Reverse: GGGTTCCATGGTGAAGTCAAC
*Il4*	Forward: ATGCACCGAGATGTTTGTACC Reverse: TTTCAGTGTTCTGAGCGTGGA
*Il6*	Forward: TCCTACCCCAACTTCCAATGCTC Reverse: TTGGATGGTCTTGGTCCTTAGCC
*Il18*	Forward: AAACCCGCCTGTGTTCGA Reverse: ATCAGTCTGGTCTGGGATTCGT
*Il33*	Forward: TCGCACCTGTGACTGAAATC Reverse: ACACAGCATGCCACAAACAT
*Tnf*	Forward: AAATGGGCTCCCTCTCATCAGTTC Reverse: TCTGCTTGGTGGTTTGCTACGAC
*Ccl2*	Forward: ATGCAGTTAATGCCCCACTC Reverse: TTCCTTATTGGGGTCAGCAC
*Ccl3*	Forward: CATGGCGCTCTGGAACGAA Reverse: TGCCGTCCATAGGAGAAGCA
*Ccl4*	Forward: TATGAGACCAGCAGCCTTTGC Reverse: GCACAGATTTGCCTGCCTTT
*Ccl5*	Forward: TGCCCACGTGAAGGAGTATTT Reverse: TTCTTCTCTGGGTTGGCACAC
*Cxcl1*	Forward: CCCCCATGGTTCAGAAGATTG Reverse: TTGTCAGAAGCCAGCGTTCAC
*Ifna*	Forward: CTGCTGTCTAGGATGTGACCTGC Reverse: TTGAGCCTTCTGGATCTGCTG
*Ifnb*	Forward: CGTTCCTGCTGTGCTTCTC Reverse: TGTAACTCTTCTCCATCTGTGAC
*Ifng*	Forward: ACGCCGCGTCTTGGTTT Reverse: AGGCTTTCAATGAGTGTGCTT
*Gmcsf*	Forward: AGACCCGCCTGAAGCTATACAA Reverse: CTGGTAGTGGCTGGCTATCATG
*Il10*	Forward: CACTGCTATGTTGCCTGCTC Reverse: TTCATGGCCTTGTAGACACC
*Tgfb*	Forward: CGTGGAAATCAATGGGATCAG Reverse: GGAAGGGTCGGTTCATGTCA

### Cytokine/chemokine generation measurement

MCs suspended in the medium for rat MCs were incubated with zymosan at final concentrations of 0.01, 0.1, 1, 10, or 50 μg/ml, resiquimod (R848) at a final concentration of 5 μg/ml (positive control for IFN assays), mouse anti-rat IgE monoclonal IgG1 antibodies at a final concentration of 5 μg/ml (positive control for GM-CSF, TNF, CCL2, and CCL3 assays), or medium alone (spontaneous cytokine/chemokine generation). Cells were incubated at 37°C in an incubator containing 5% CO_2_ humidified atmosphere for 3 h. Afterward, the cell suspensions were centrifuged (150×*g*, 5 min, 20°C), and the supernatants were transferred directly into a new set of tubes. Commercial ELISA test kits measured cytokine/chemokine levels according to the manufacturers’ instructions. The sensitivity of IFN-α, IFN-β, IFN-γ, GM-CSF, TNF, CCL2, and CCL3 test was <31.2 pg/ml, <7.8 pg/ml, <7 pg/ml, <15 pg/ml, <9.375 pg/ml, <10 pg/ml, and <15 pg/ml, respectively.

### ROS generation measurement

Native and IgE-coated MCs suspended in the medium for rat MCs were incubated with zymosan at final concentrations of 0.01, 0.1, 1, 10, or 50 μg/ml, curdlan at a final concentration of 50 μg/ml (positive control), or with medium alone (spontaneous ROS generation). Incubation was carried out for 2 h at 37°C in an incubator containing 5% CO_2_ humidified atmosphere. Afterward, CellROX® Reagent at a final concentration of 5 μM was added, and cells were incubated at 37°C for 30 min. Then, the triple-wash procedure of cells in 1 × PBS was carried out by centrifugation (150×*g*, 5 min, 20°C). Fluorescence intensity was measured using FLUOstar Omega Microplate Reader (BMG Labtech) with an excitation wavelength of 485 nm and an emission wavelength of 520 nm. ROS level was expressed as an MSI (mean signal intensity).

### Treatment of MCs with signaling pathway inhibitors

To analyze the involvement of signaling molecules in zymosan-induced response, purified MCs were pretreated with a Syk kinase inhibitor R406 at a concentration of 2 μM, Dectin-1 antagonist, i.e., laminarin at a concentration of 50 μg/ml, or medium alone at 37°C in an incubator containing 5% CO_2_ humidified atmosphere for 30 min, before the main procedure was carried out. The used concentrations of R406 and laminarin were chosen in the preliminary experiments, and none of them affected MC viability, as assessed by the trypan blue dye exclusion assay.

### Statistical analysis

Statistical data analysis was performed using STATISTICA 13 software (Statsoft Inc., Tulsa, OK). Results were presented as mean ± standard deviation (SD). The normality of distribution was evaluated using the Shapiro-Wilk test. Student’s *t*-test evaluated significant differences for small groups (a value of *P* < 0.05 was considered significant).

## Results

### The effect of zymosan on MC migration

**Fig. 1:**
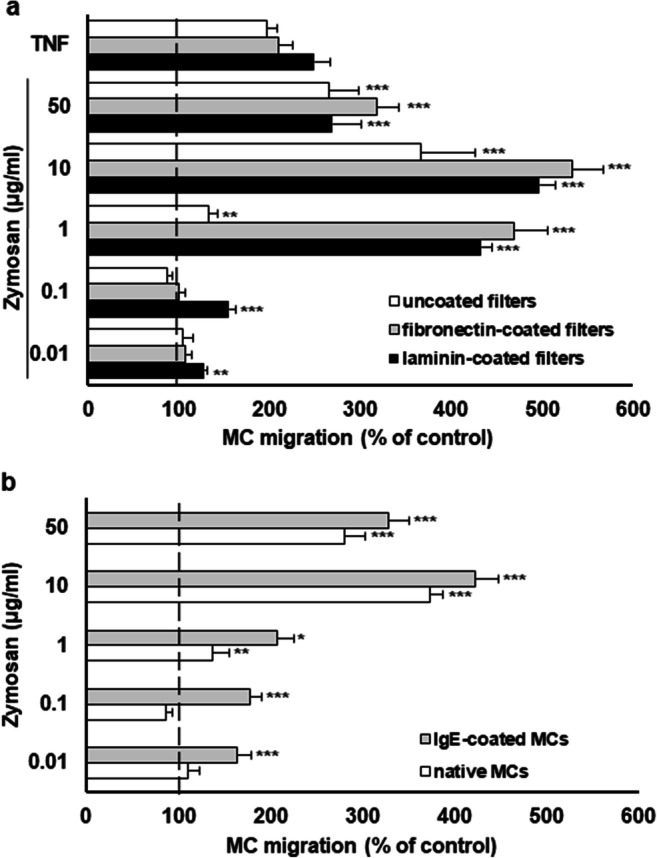
Zymosan-induced MC migration. (**a**) Native MCs were incubated with different zymosan concentrations, TNF at 0.05 pg/ml (positive control), or medium alone (control spontaneous MC migration) for 3 h in a Boyden microchamber. Uncoated (white bars), fibronectin-coated (gray bars), or laminin-coated (black bars) filters were used. (**b**) Native and IgE-coated MCs were incubated with different concentrations of zymosan or medium alone (control spontaneous MC migration) for 3 h in a Boyden microchamber using uncoated filters. Ten HPF were counted in each assay (×250). Spontaneous cell migration served as a control and was identified to be 100%. Results are shown as the mean ± SD of three independent experiments, and each experiment was carried out in duplicate samples (each experiment was performed on 2 animals/MC isolates). *P < 0.05, **P < 0.01, ***P < 0.001

The influence of zymosan on MC migration was also examined. To this end, the upper wells of the 48-well Boyden microchamber were filled with MC suspension and separated from the lower wells, where various concentrations of zymosan, TNF (positive control), or medium alone (control spontaneous MC migration) were applied, by 8-μm-pore-sized polycarbonate filters. We found that zymosan at concentrations from 1 to 50 μg/ml induced migratory response of native MCs (Fig. 1a). The optimal concentration of zymosan for MCs’ maximal migration was 10 μg/ml (reaching 366.8 ± 60.4% of control spontaneous migration). We also stated that the coating of the filters with ECM proteins, i.e., laminin and fibronectin, enhanced MC migration induced by zymosan. Interestingly, pretreatment of MCs with 1 μg/ml of IgE strongly augmented migration towards zymosan at all concentrations used (Fig. 1b). Statistical analysis also revealed that migration of IgE-primed cells was significantly higher than that for non-primed cells in response to 0.01 μg/ml (*P* < 0.001), 0.1 μg/ml (*P* < 0.001), 1 μg/ml (*P* < 0.001), 10 μg/ml (*P* < 0.001), and 50 μg/ml (*P* < 0.001) of zymosan.

The zymosan ability to stimulate directional migration (chemotaxis) or random migration (chemokinesis) was tested using a checkerboard analysis. We observed that MCs’ zymosan-induced migration is gradient dependent, and thus chemotaxis occurred (Table 2).

**Table 2:** Checkerboard analysis of zymosan-induced MC migratory response

Zymosan in the bottom well (μg/ml)	Zymosan in top well (μg/ml)			
	0	1	10	50
0	100 ± 6	176 ± 25	506 ± 44	682 ± 21
1	139 ± 10**	435 ± 33	646 ± 42	366 ± 13
10	376 ± 14***	368 ± 13	330 ± 18	295 ± 27
50	286 ± 12***	292 ± 24	513 ± 25	410 ± 36

MCs were stimulated with various concentrations of zymosan or medium alone (control spontaneous MC migration) for 3 h at 37°C in a Boyden microchamber. Ten HPF were counted in each assay (×250). Spontaneous cell migration served as a control and was identified to be 100%. Results are shown as the mean ± SD of three independent experiments, and each experiment was carried out in duplicate samples (each experiment was performed on 2 animals/MC isolates). **P < 0.01, ***P < 0.001

### The effect of zymosan on MC histamine release and cysLT generation

**Fig. 2:**
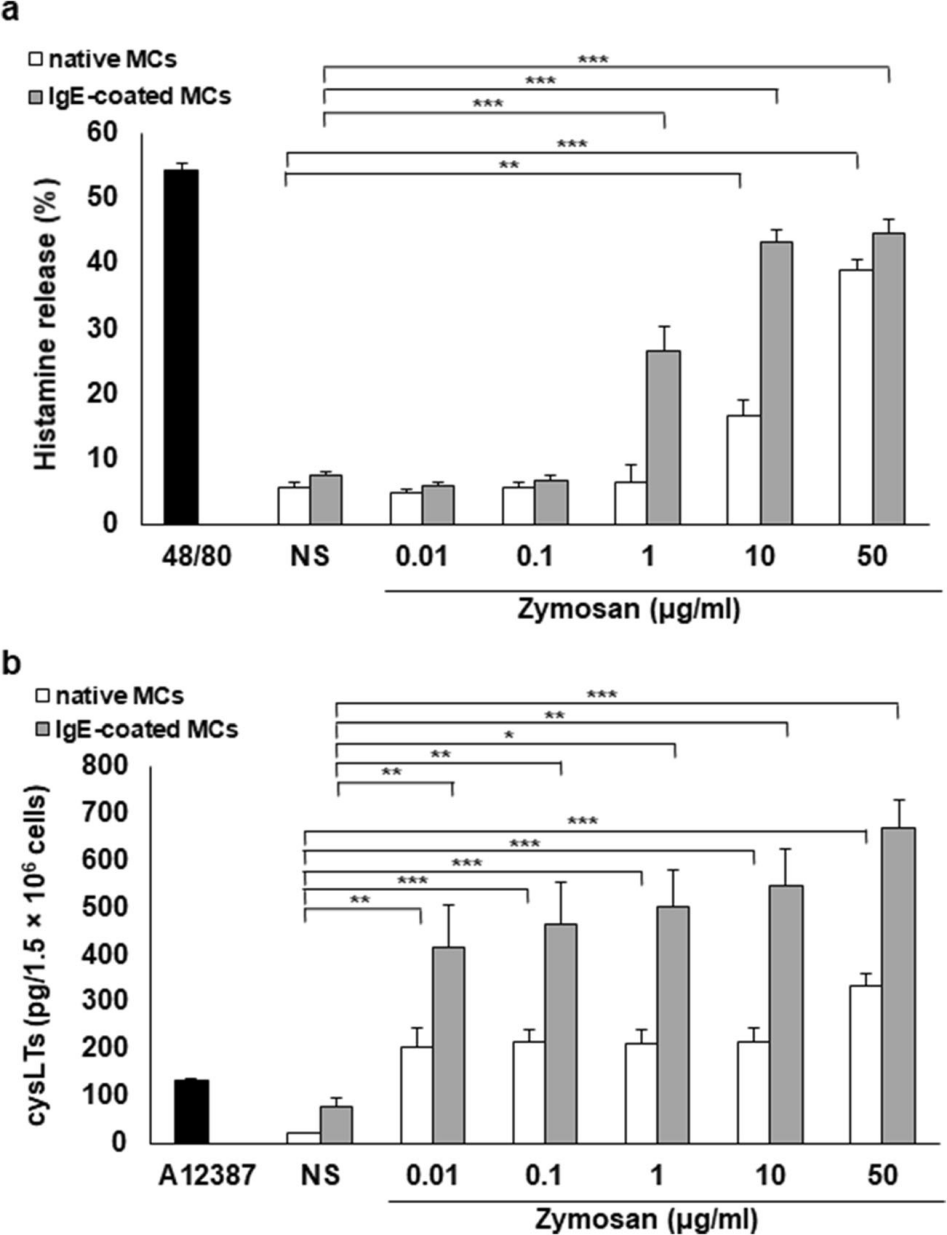
Zymosan-induced histamine release (**a**) and cysLT synthesis (**b**) by MCs. (**a**) Native and IgE-coated MCs were stimulated with various concentrations of zymosan, compound 48/80 at 5 μg/ml (positive control), or medium alone (NS) for 30 min. (**b**) Native and IgE-coated MCs were incubated with various concentrations of zymosan, A23187 at 5 μg/ml (positive control), or medium alone (NS) for 1 h. Results are shown as the mean ± SD of three independent experiments, and each experiment was carried out in duplicate samples (each experiment was performed on 2 animals/MC isolates). *P < 0.05, **P < 0.01, ***P < 0.001

We first examined the zymosan ability, used in a range of concentrations from 0.01 to 50 μg/ml, to direct stimulation of native and IgE-coated MCs to degranulation and histamine release. We found that zymosan noticeably activated native MCs to secrete histamine only in response to higher concentrations used, i.e., 10 and 50 μg/ml (Fig. 2a). For comparison, at the same experimental conditions, native MCs released up to 54.3 ± 1.8% of histamine to the challenge with compound 48/80, the well-known MC degranulating agent [23]. We also noted an enhanced histamine secretion from IgE-sensitized MCs than from non-stimulated IgE-primed MCs upon exposure to 1–50 μg/ml zymosan. Furthermore, statistical analysis revealed that IgE-coated MCs released significantly higher amounts of histamine than native cells in response to 1 μg/ml (26.6 ± 3.7% versus 6.6 ± 2.6%, *P* < 0.001), 10 μg/ml (43.2 ± 2.1% versus 16.6 ± 2.6%, *P* < 0.001), and 50 μg/ml (44.5 ± 2.3% versus 38.9 ± 1.5%, *P* < 0.001) of zymosan. To verify the time dependence of histamine release, MCs were stimulated with zymosan at a concentration of 50 μg/ml. We observed a slight histamine release within 3 min following incubation with zymosan. After 5 min of exposure, zymosan-induced histamine secretion from native MCs was statistically significant (*P* < 0.001) (data not shown).

Next, we investigated whether zymosan could directly activate both native and IgE-primed MCs to produce and release cysLTs. As demonstrated in Fig. 2b, zymosan, at all tested concentrations, noticeably induced synthesis of these mediators by MCs. MCs’ maximal cysLT generation (333.9 ± 28.2 pg/1.5 × 10^6^ cells) was observed when zymosan was used at a concentration of 50 μg/ml. In comparison, MCs treated with calcium ionophore A12387, i.e., a well-documented inducer of LTs generation [24], released up to 133.7 ± 4.1 pg/1.5 × 10^6^ cells of cysLTs. Interestingly, MCs primed with 1 μg/ml of IgE secreted considerably higher amounts of cysLTs in response to all zymosan concentrations used as compared to non-stimulated IgE-sensitized MCs. We also noticed a significant increase in cysLT release from IgE-coated MCs as compared to non-primed cells challenged with zymosan at a concentration of 0.01 μg/ml (416.7 ± 91.1 versus 204.0 ± 40.1 pg/1.5 × 10^6^ cells; *P* < 0.05), 0.1 μg/ml (463.3 ± 91.3 versus 214.8 ± 25.4 pg/1.5 × 10^6^ cells; *P* < 0.05), 1 μg/ml (501.6 ± 79.3 versus 212.2 ± 30.3 pg/1.5 × 10^6^ cells; *P* < 0.01), 10 μg/ml (545.6 ± 78.2 versus 215.1 ± 31.0 pg/1.5 × 10^6^ cells; *P* < 0.01), and 50 μg/ml (670.1 ± 57.8 versus 333.9 ± 28.2 pg/1.5 × 10^6^ cells; *P* < 0.01).

### The effect of zymosan on mRNA cytokine and chemokine expression

**Fig. 3:**
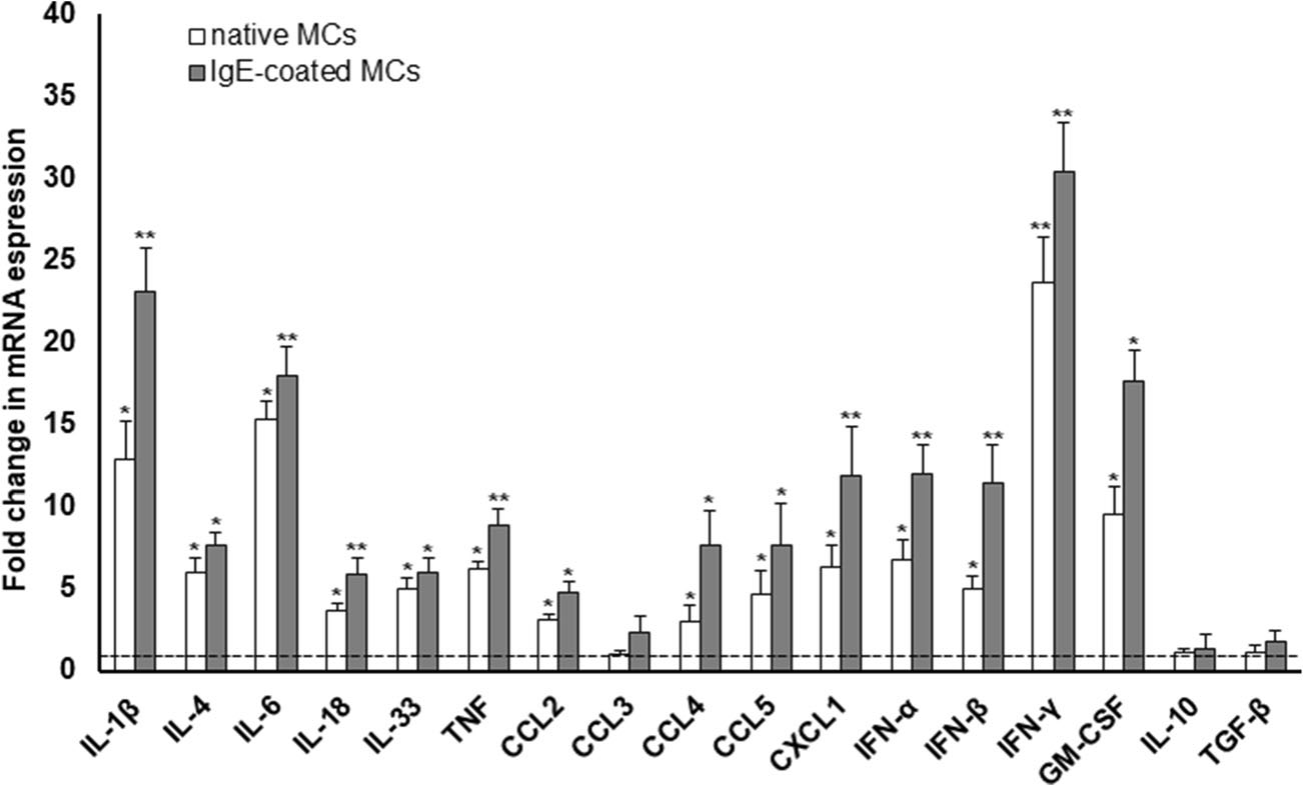
Zymosan-mediated cytokine/chemokine mRNA transcript levels in MCs. Native and IgE-coated MCs were stimulated with zymosan at 50 μg/ml for 2 h. Total RNA was isolated and converted into cDNA, and qRT-PCR was conducted to assess cytokine/chemokine mRNA levels. The expression of mRNAs was corrected by normalization based on the transcript level of the reference gene rat *Actb*. Results are shown as the mean ± SD of three independent experiments, and each experiment was carried out in duplicate samples (each experiment was performed on 2 animals/MC isolates). *P < 0.05, **P < 0.01

Then, we assessed the fold change in mRNA expression of certain cytokines and chemokines in zymosan-stimulated MCs (50 μg/ml) compared to non-stimulated cells (Fig. 3). Exposure of MCs with zymosan caused most markedly increase for IFN-γ (23.6-fold), IL-6 (15.3-fold), IL-1β (13-fold), and GM-CSF (9.6-fold) mRNA levels. We also noticed augmented transcript levels of IFN-α (6.8-fold), TNF (6.3-fold), CXCL1 (6.3-fold), IL-4 (6.1-fold), IL-33 (5.1-fold), IFN-β (5.0-fold), and CCL5 (4.7-fold). A slight increase in the mRNA expression level of IL-18 (3.7-fold), CCL2 (3.2-fold), and CCL4 (3.1-fold) was also observed. Concomitantly, we found that zymosan did not influence TGF-β, CCL3, and IL-10 transcript expression. Additionally, we noted that MC sensitization with IgE notably enhanced mRNA expression levels of studied cytokines and chemokines. MC priming with IgE resulted in an increase of zymosan-induced mRNA level for IFN-γ (30.4-fold), IL-1β (23.1-fold), IL-6 (18.0-fold), GM-CSF (17.6-fold), IFN-α (12.1-fold), CXCL1 (12.0-fold), IFN-β (11.5-fold), TNF (8.9-fold), IL-4 (7.7-fold), CCL4 (7.7-fold), CCL5 (7.6-fold), IL-33 (6.1-fold), IL-18 (5.9-fold), and CCL2 (4.8-fold) in relative to non-stimulated IgE-primed MCs. Also, the mRNA level of CCL3 was 2.4-fold increased in IgE-primed MCs. However, even after MC sensitization with IgE, we did not observe changes in TGF-β and IL-10 and mRNA transcript levels in response to zymosan.

### The effect of zymosan on MC cytokine and chemokine generation

**Fig. 4:**
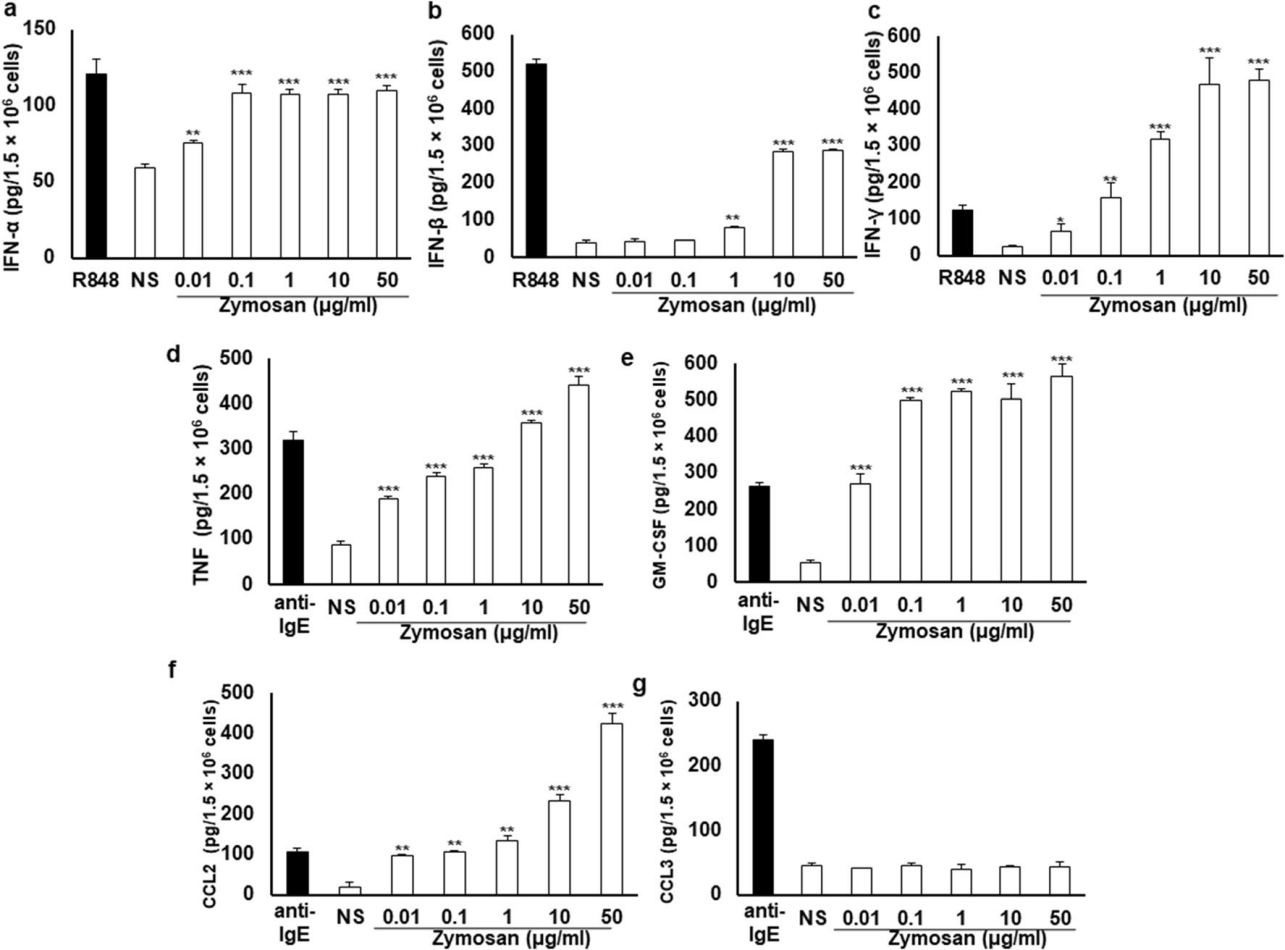
Zymosan-induced IFN-α (**a**), IFN-β (**b**), IFN-γ (**c**), TNF (**d**), GM-CSF (**e**), CCL2 (**f**), and CCL3 (**g**) synthesis by MCs. Native MCs were stimulated with various concentrations of zymosan, R848 at 5 μg/ml (positive control for IFNs assays) or anti-IgE at 5 μg/ml (positive control for TNF, GM-CSF, CCL2, and CCL3 assays), or medium alone (NS) for 3 h. Results are shown as the mean ± SD of three independent experiments, and each experiment was carried out in duplicate samples (each experiment was performed on 2 animals/MC isolates). **P < 0.01, ***P < 0.001

Afterward, we studied if zymosan has the capability to induce IFN-α, IFN-β, and IFN-γ synthesis by MCs. In comparison, we assessed the level of IFNs generated by MCs stimulated with R848, a well-recognized synthetic mimic of viral ssRNA and TLR7 agonist [25]. As presented in Fig. 4a–c, treatment with zymosan activated MCs to generate all studied IFNs. Zymosan in the range of concentrations from 0.1 to 50 μg/ml caused a considerable IFN-α production, comparable to R848-activated IFN-α synthesis. Of the various concentrations of zymosan, the greatest IFN-β generation was observed at 10 μg/ml (283.6 ± 6.0 pg/1.5 × 10^6^ cells) and 50 μg/ml (287.8 ± 2.9 pg/1.5 × 10^6^ cells). IFN-γ generation and release in response to MC stimulation with zymosan at a concentration of 1, 10, and 50 μg/ml resulted in almost 2.5-, 3.7-, and 4-fold higher IFN-γ generation than that induced by R848, respectively.

Additionally, we found that zymosan stimulated MCs to dose-dependent TNF generation at all tested concentrations (Fig. 4d). As shown in Fig. 4e, more significant GM-CSF amounts were synthesized and released by MCs challenged with zymosan in the entire range of concentrations used than those stimulated with anti-IgE. As presented in Fig. 4 f and g, zymosan activated MCs to generate CCL2, but not CCL3. After MC treatment with zymosan at 0.01–1 μg/ml, CCL2 generation was comparable to anti-IgE-induced synthesis. In turn, the greatest CCL2 generation was observed at 50 μg/ml of zymosan, rising to 425.3 ± 24.3 pg/1.5 × 10^6^ cells.

### The effect of zymosan on ROS generation by MCs

**Fig. 5:**
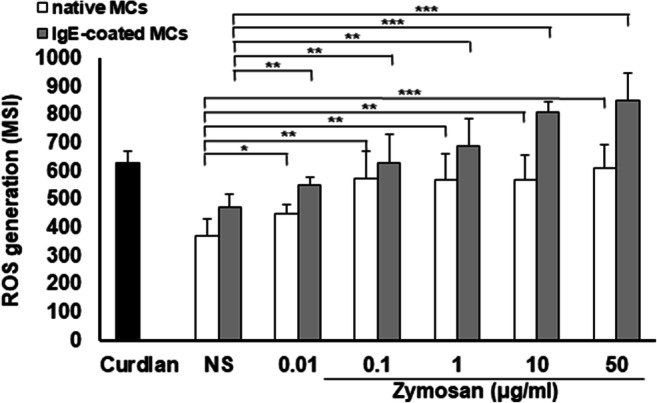
Zymosan-induced ROS generation by MCs. Native and IgE-coated MCs were stimulated with various zymosan concentrations, curdlan at 50 μg/ml (positive control), or medium alone (NS) for 1 h. CellROX™ Green Reagent was used at a concentration of 5 μM for 30 min. Results are shown as the mean ± SD of three independent experiments, and each experiment was carried out in duplicate samples (each experiment was performed on 2 animals/MC isolates). *P < 0.05, **P < 0.01, ***P < 0.001

We were also interested in whether zymosan activates native as well as IgE-primed MCs to generate ROS. For comparison, ROS production by MCs in response to activation with curdlan [26] was determined. As demonstrated in Fig. 5, stimulation of native MCs with zymosan significantly induced ROS production relative to non-stimulated MCs. ROS generation in response to activation with 50 μg/ml of zymosan was comparable to curdlan-induced ROS level. Moreover, we found that MC sensitization notably enhanced ROS generation’s potential compared to non-stimulated IgE-primed cells. Statistical analysis also indicated that ROS production by IgE-primed MCs was significantly higher than that by non-primed MCs in response to zymosan stimulation at 0.01 μg/ml (550.2 ± 28.6 versus 451.8 ± 29.6 MSI, *P* < 0.001), 10 μg/ml (808.2 ± 40.6 versus 571.2 ± 88.4 MSI, *P* < 0.001), and 50 μg/ml (852.2 ± 96.4 versus 611.2 ± 85.0 MSI, *P* < 0.01).

### The effect of laminarin and Syk inhibitor on zymosan-mediated MC response

**Fig. 6:**
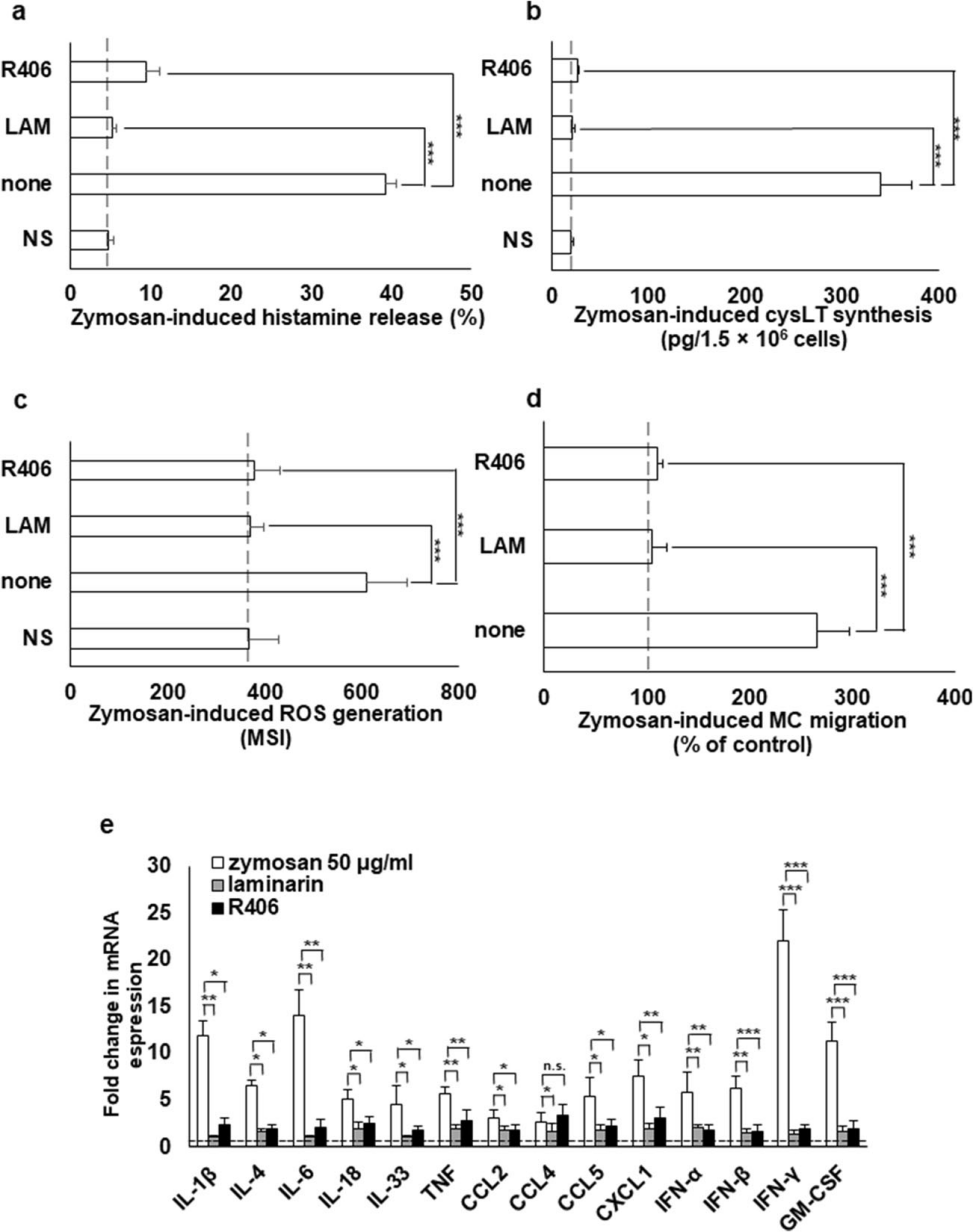
Effect of Syk inhibitor R406 and Dectin-1 receptor antagonist laminarin on zymosan-induced MC histamine release (**a**), cysLT synthesis (**b**), ROS generation (**c**), migration (**d**), and cytokine/chemokine mRNA expression (**e**). MCs were preincubated with Syk inhibitor R406 at 2 μM, laminarin at 50 μg/ml, or medium alone (NS) for 30 min before stimulation with zymosan at 50 μg/ml. (**a**–**d**) Results are shown as the mean ± SD of three independent experiments, and each experiment was carried out in duplicate samples (each experiment was performed on 2 animals/MC isolates). ***P < 0.001. (**e**) Total RNA was isolated and converted into cDNA, and qRT-PCR was conducted to assess cytokine/chemokine mRNA levels. The expression of mRNAs was corrected by normalization based on the transcript level of the reference gene rat *Actb*. Results are shown as the mean ± SD of three independent experiments, and each experiment was carried out in duplicate samples (each experiment was performed on 2 animals/MC isolates). *P < 0.05, **P < 0.01, n.s., non significant

To examine whether the Dectin-1 receptor is involved in zymosan-mediated MC response, laminarin, Dectin-1 receptor antagonist, and R406, Syk kinase inhibitor were used. As shown in Fig. 6a, preincubation of MCs with laminarin and R406 completely abolished (*P* < 0.001) zymosan-mediated histamine release from 39.3 ± 1.4% to 5.1 ± 0.6% and 9.5 ± 1.6%, respectively. Likewise, we observed that following MC preincubation with laminarin as well as R406, zymosan-induced cysLT synthesis, and release was significantly reduced (*P* < 0.001) (Fig. 6b). We also established that laminarin and R406 completely (*P* < 0.001) inhibited zymosan-induced ROS generation by MCs (Fig. 6c). Pretreatment of MC with laminarin and R406 totally abrogated cell migration in response to zymosan from 265.3 ± 32.2% to 104.4 ± 14.3% and 110.5 ± 4.4% of control migration, respectively (Fig. 6d). MC pretreatment with the laminarin and R406 led to a noticeable decrease in the zymosan-mediated mRNA expression level of cytokines and chemokines, including IL-1β, IL-4, IL-6, IL-18, IL-33, TNF, CCL2, CCL4, CCL5, CXCL1, IFN-α, IFN-β, IFN-γ, and GM-CSF (Fig. 6e).

## Discussion

There is a lot of data indicating that bacteria-derived components affect various aspects of MC biology, including their phenotype, degranulation, generation and/or release of mediators, survival, or migration [27, 28]. Also, the effect of viral molecules on MC activity becomes well recognized [10, 29]. In turn, very little is known about the influence of fungi constituents on the MC response. The major fungi molecules that affect cell response include primarily structural components of their cell wall. In the presented study, we were interested whether zymosan, i.e., β-(1,3)-glucan containing mannan particles, affects the activity of fully mature MCs. Throughout all experiments, we used zymosan extracted from *S. cerevisiae* cell wall, which is commonly served as a model fungal molecule to study interactions with host immune cells [19–22, 30].

Overall, our results directly indicate that zymosan has the potential to elicit a pro-inflammatory response of MCs. Most importantly, this is the first study to reveal that zymosan can induce MC migration, even in the absence of ECM proteins. It means that zymosan may constitute a potent chemoattractant for MCs, and this finding is of great significance. Moreover, concerning MC motility characteristics, we documented that the zymosan-induced migratory response of these cells was almost entirely a result of directional migration, i.e., chemotaxis. It should be stressed that in the case of physiological conditions, the MC number in tissues is kept relatively constant but increases remarkably during inflammation. Thus, our observation is highly relevant as it may indicate that MCs accumulate in large numbers at the site of fungi infection. In turn, the accumulation of MCs at the inflamed milieu leads to further exacerbation and amplification of the ongoing inflammatory process [6].

We also found that zymosan induces MCs’ degranulation as assessed by histamine release and stimulated those cells to generate lipid metabolites, that is, cysLTs. It is noteworthy that histamine and cysLTs are versatile mediators of inflammation as they affect vascular permeability or cell adhesion to the vascular epithelium. Our observations suggest that by producing those mediators, MCs may have the potency to exert further pro-inflammatory cascades and recruit immune cells, e.g., neutrophils and monocytes/macrophages to the site of a fungi infection [31, 32]. Previously, the effect of zymosan on cysLT production by MCs was demonstrated only on CBMC model [19, 20]. We herein also reported that zymosan stimulates MCs to the generation and release of pro-inflammatory/immunoregulatory cytokines/chemokines as well as it notably increases their mRNA expression. At the same time, we did not observe changes in the mRNA expression level of anti-inflammatory cytokines, and this finding is of considerable interest. Earlier studies demonstrated the zymosan-induced synthesis of IL-1β and GM-CSF protein only by CBMCs [19, 20]. Here, we observed that stimulation of MCs with both low and high concentrations of zymosan leads to TNF, GM-CSF, IFN-α, IFN-γ, and CCL2 generation, whereas IFN-β was released only upon MC treatment with high doses of this fungi-derived constituent. This phenomenon is of considerable interest. It might be hypothesized that stimulation of MCs with various doses of zymosan can result in differences in the affinity, avidity, or threshold of signaling via Dectin-1 and that this translates into qualitative changes in the signaling pathways with the involvement of interferon regulatory factor (IRF)3, which is crucial for IFN-β synthesis [33]. Emerging data highlight multidirectional role of studied cytokines/chemokines in the immune response against some fungal pathogens [34, 35]. They are critical in mediating host cell-to-cell communication and promoting the initiation, maintenance, or resolution of the inflammatory processes during infection. Therefore, we postulate that the activation and trafficking of immune cells involving in antifungal defense, such as neutrophils, monocytes/macrophages, dendritic cells, or various T cell subpopulations to the site of fungal infection, may be likely dependent on MC-released cytokines and chemokines. Of great interest, MC-derived mediators may play an important role in the differentiation and functions of Th17 lymphocytes, a cell subset that has a potent role in bridging the adaptive and the innate immune response against fungi [36, 37]. For instance, Th17 cell differentiation from naïve T cells can be initiated by IL-6 and is further reinforced by IL-23 [38, 39]. There exists evidence that cysLTs or chemokine CCL2 act as chemoattractants for Th17 cells and therefore may regulate their infiltration to the infected tissue [40, 41]. Since Th17 cells express functional histamine H4 receptor, it can be also stated that MC-derived histamine may affect Th17 activity [42]. In turn, it is worthy to note that Th17 cells constitute a source of IL-17 and IL-22, which are also important cytokines for the successful antifungal response [37, 43]. Additionally, we present evidence that zymosan stimulated mature MCs to generate ROS, and this finding is in line with the previous reports documenting the zymosan-mediated reactive free radicals generation by immature BMMCs [21, 22]. It should be stressed that ROS are incredibly harmful to microorganisms, especially at high concentrations. These factors are highly toxic to pathogenic fungi, causing damage to their DNA, proteins, or lipids and ultimately leading to their cells’ death [44]. Besides, data indicate that ROS are also essential for distinct biological functions, including immune cell survival, cell growth, proliferation, and differentiation [45]. Thus, it appears that MC-derived ROS may have an important role in the immune response and defense mechanisms against pathogenic fungi.

The initiation of the host immune response against invading fungi depends on recognizing their components by specific PRRs. Among them, Dectin-1 from the CLR group is the best-characterized PRR, which detects the β-glucans of the fungal cell wall [46]. The presence of Dectin-1 was confirmed in numerous immune cells, i.e., monocytes/macrophages, neutrophils, eosinophils, basophils, NK cells, fibroblasts, dendritic cells, B cells, and some T cell subsets [47–49]. Interestingly, the Dectin-1 expression was also documented in various MCs, including CBMCs [20], BMMCs [21], RBL-2H3 cells [50], and progenitor-derived MCs [51]. We have recently established that rat mature peritoneal MCs constitutively express a functional Dectin-1 [26]. Here, we provide evidence demonstrating that zymosan-mediated MC response is induced via activation of the Dectin-1 receptor. After MC pretreatment with laminarin, a Dectin-1 antagonist commonly used in research to prove the role of such receptor in cell activation [20, 21, 52], there was a total inhibition or significant suppression of zymosan-mediated response. Furthermore, our data suggest a critical role for Syk kinase activation after zymosan-mediated signaling through Dectin-1. We showed that inhibition of Syk using a competitive inhibitor of this molecule significantly abrogated the studied activities of MCs in response to zymosan. It should be emphasized that the Syk kinase is deeply involved in the signal transduction cascade associated with the activation of Dectin-1 [53].

Several sources suggest that the binding of monomeric IgE to FcεRI on MCs induces a sensitization process that further impacts various aspects of MC functioning [54]. It was documented that after the sensitization phase, IgE caused an increase of FcεRI surface expression as well as enhanced MC survival, histamine and leukotriene release, cytokine/chemokine generation, adhesion to the ECM proteins, and migration [54]. Moreover, it was reported that IgE-sensitization potentiated MC response to certain stimuli, such as compound 48/80 and substance P [55]. We also previously established that spontaneous and TNF- or CCL5-induced migratory response of IgE-coated MCs was essentially higher than the migration of native cells [56]. To date, only two reports suggest that IgE sensitization may affect MC responsiveness to fungi particles. Selander et al. [57] revealed that *Malassezia sympodialis* extract enhanced IL-6 production by IgE-sensitized BMMCs. We have recently found that also mannan from *S. cerevisiae* strengthened some aspects of IgE-sensitized MC activity [58]. Thus, in the presented paper, we wished to determine if priming of MCs with IgE may alter their responsiveness to zymosan. We stated, for the first time, that IgE-primed MCs were substantially more responsive to activation with zymosan, which we noticed for histamine release, cysLT synthesis, cytokine/chemokine mRNA transcript expression, ROS generation, and migration. This finding would seem to imply that fungal infection, similar to bacterial and viral infections [59, 60], could exacerbate and/or worsen the course of IgE-dependent disorders including allergic ones.

Some earlier studies have shown the effects of other fungi-associated PAMPs on MC activity. It was documented that β-glucan and mannan derived from *Candida albicans* induced RBL-2H3 cell degranulation and β-hexosaminidase secretion [61]. Also, our recent evidence indicates that mannan obtained from *S. cerevisiae* activated peritoneal MCs to degranulate and generate several pro-inflammatory/immunoregulatory mediators and ROS as well as serve as a potent chemoattractant for these cells [58]. In contrast, β-(1,3-1,6)-glucan isolated from *Aureobasidium pullulans* inhibited the degranulation of both BMMCs and RBL-2H3 cells [62]. There exists evidence that curdlan, a purified linear fungal-like β-(1,3)-glucan extracted from bacterium *Alcaligenes faecalis*, also impacts MC activity. Kimura et al. [50] documented that curdlan induced mRNA expression of IL-3, IL-4, IL-13, TNF, and CCL2 in RBL-2H3 cells. Additionally, it was demonstrated that curdlan activated peritoneal MCs to migrate and synthesize mediators, such as histamine, cysLTs, IFN-α, IFN-γ, GM-CSF, TNF, CCL3, and ROS [26].

Evidence for MC involvement in the immune response to microbial pathogens continues to grow. The findings presented in this paper show that mature peritoneal MCs respond to fungal zymosan via Dectin-1. We noted that activation with zymosan results in the synthesis of pro-inflammatory and immunoregulatory mediators and ROS; thus, present findings confirm those obtained for BMMC or CBMC model [19–22]. In turn, our observation that zymosan promoted MC chemotaxis is entirely novel. Additionally, we report for the first time that MC sensitization with IgE amplified their activity upon exposure to zymosan. Overall, our results strongly support the notion that MCs contribute to innate antifungal immunity and bring us closer to elucidate their role in host-pathogenic fungi interactions.
